# Absorption, translocation, and effects of Bt Cry1Ac peptides from transgenic cotton to the intercrops and soil functional bacteria

**DOI:** 10.1038/s41598-020-73375-8

**Published:** 2020-10-14

**Authors:** Wei Zhang, Zhen Cao, Mian Wang, Xiaojiao Chen, Baomin Wang

**Affiliations:** College of Agriculture and Biotechnology, China Agricultural University, Yuanmingyuan west road No.2, Beijing, 100193 China

**Keywords:** Biochemistry, Biotechnology

## Abstract

Insecticidal proteins encoded by the truncated genes from *Bacillus thuringiensis* (*Bt*) in transgenic crops are released into soil mainly through root exudate and crop residues. In the present study, Bt Cry1Ac protein was hydrolyzed by pronase that was secreted by the soil bacterium *Streptomyces griseus*. Six peptides were identified as the products of enzymatic hydrolysis by nano liquid chromatography tandem mass spectrometry (LC–MS/MS). One of the six peptides was labeled with radioactive isotope iodine-125 and then purified. The ^125^I-peptide solution was irrigated to the rhizosphere soil of watermelon seedlings (*Citrullus lanatus* L.) and wheat seedlings (*Triticum aestivum* L.), which the two crops usually intercrop with cotton in China. Detection of radioactivity in both plant tissues within one hour proved adsorption, uptake and translocation of the peptide into watermelon and wheat seedlings. Three of the identified peptides were sprayed onto the seedling leaves of watermelon, wheat and maize (*Zea mays* L.) in the field or the growth chamber. No significant effects on plant growth were observed. These peptides also did not affect growth of organic phosphate-dissolving, nitrogen-fixing, and potassium-dissolving bacteria in the culture. This study provides a new view of GMO risk assessment methodology.

## Introduction

Bt cotton, producing *Bacillus thuringiensis* (Bt) toxins and thus reducing insecticide usage^1^, has been widely cultivated around the world. Bt protein toxins are released into soil from root exudate^2^, decayed biomass^3^, pollens^4^ and animal manures^5^. The repeated and large-scale cultivation of Bt crops could result in the accumulation of Bt protein in soil^6^. The studies of Bt protein accumulation in soil have been mainly focused on the content and half-life^7,8^. The concentrations of Bt protein have been detected in a range from 5 to 100 ng/g^9^ or from 200 to 300 ng/g in soil^10^. Head et al.^7^ estimated that plant biomasses contribute at least 650 ng/g of Cry1Ac protein in a Bollgard^®^ cotton field. Cry1Ab toxin concentrations decrease to 38% of the initial concentration after 40 days and to 0.3% in 200 days^3^. The degradation half-life (DT_50_) of Bt protein is approximately 240 days in the tissues of transgenic corn, cotton and potato^11^. Moreover, Bt proteins bond rapidly and tightly to clays and organic matters in soil^6,12^. Such binding reduces the biodegradability and increases the persistence of Bt proteins^12,13^. Therefore, Bt proteins may remain biological activities for over hundreds of days in soil.

Numerous studies have been reported the effects of different Bt crops on microbes^6,14–17^, algae^6^, earthworms^6,18^, terrestrial isopods^19^, nematodes^6,16,20^, protozoa^6,16^, corn rootworms^20^ and arthropods^20,21^ in soils. The bacterial communities and mycorrhizal establishment are lower obviously in the Bt-corn planted field^14^. The pre-symbiotic hyphal growth in root exudates and appressoria of Bt 176 transgenic maize are significantly decreased in comparison with the non-transgenic plants^22^. However, a majority of the studies indicated that Bt proteins have no or negligible impact on soil ecosystem^15–17,19,20,23^. The Bt toxins from three subspecies including *Bacillus thuringiensis* subsp. Kurstaki (Btk)*, B. thuringiensis* subsp. Morrisoni strain Tenebrionis (Btt) and *B. thuringiensis* subsp. Israelensis (Bti) did not inhibit the growth of bacteria, fungi, yeasts, algae and cyanobacterium^17^. The soil planted with Bt corn did not show the change in the numbers of some soil mites, collembola, and nematodes^20^. Under field conditions, there is little difference in microflora between Bt-potato plants and commercial potato plants^15^. The laboratory studies showed little adverse effects of Cry1Ab transgenic maize leaves on *Trachelipus rathkii* and *Armadillidium nasatum*^19^. The different effects may be related to Bt protein content and bacterial community.

In 2015, China planted about 3.7 million hectares of Bt-cotton, being 96% of its 3.8 million total cotton acreage^24^. Cotton-based intercropping, such as wheat-cotton, is a common practice^25^. Cotton is often intercropped with peanut, soybean, chickpea, onion, radish, chills^26^, watermelon^27^, jujube and apricot. Microorganisms in soil can utilize free and clay-bound Bt proteins as the carbon source and energy^28–30^. Bt proteins can be enzymatically digested, such as by bacterial and fungal proteases, into peptides and degraded proteins^31^. *Streptomyces griseus*, for example, can produce the proteolytic enzyme pronase^32^. Effects of peptides derived from Bt protein on soil bacteria remain unclear. In field circumstances, Bt proteins are degraded into small peptides or free amino acids. The uptake of the Bt protein by various crops from soils on which Bt corn had previously grown was reported^33,34^. However, few studies on absorption, uptake and translocation of metabolic peptides of Bt proteins into intercrops were conducted. In the present study, we identified some peptides of Bt protein hydrolyzed by soil bacteria secreted enzymes, studied the uptake and translocation of the peptides to other crops, and investigated toxic effects of these peptides on soil bacteria.

## Results

### Enzymolysis of Bt Cry1Ac and identification of peptides

**Figure 1:**
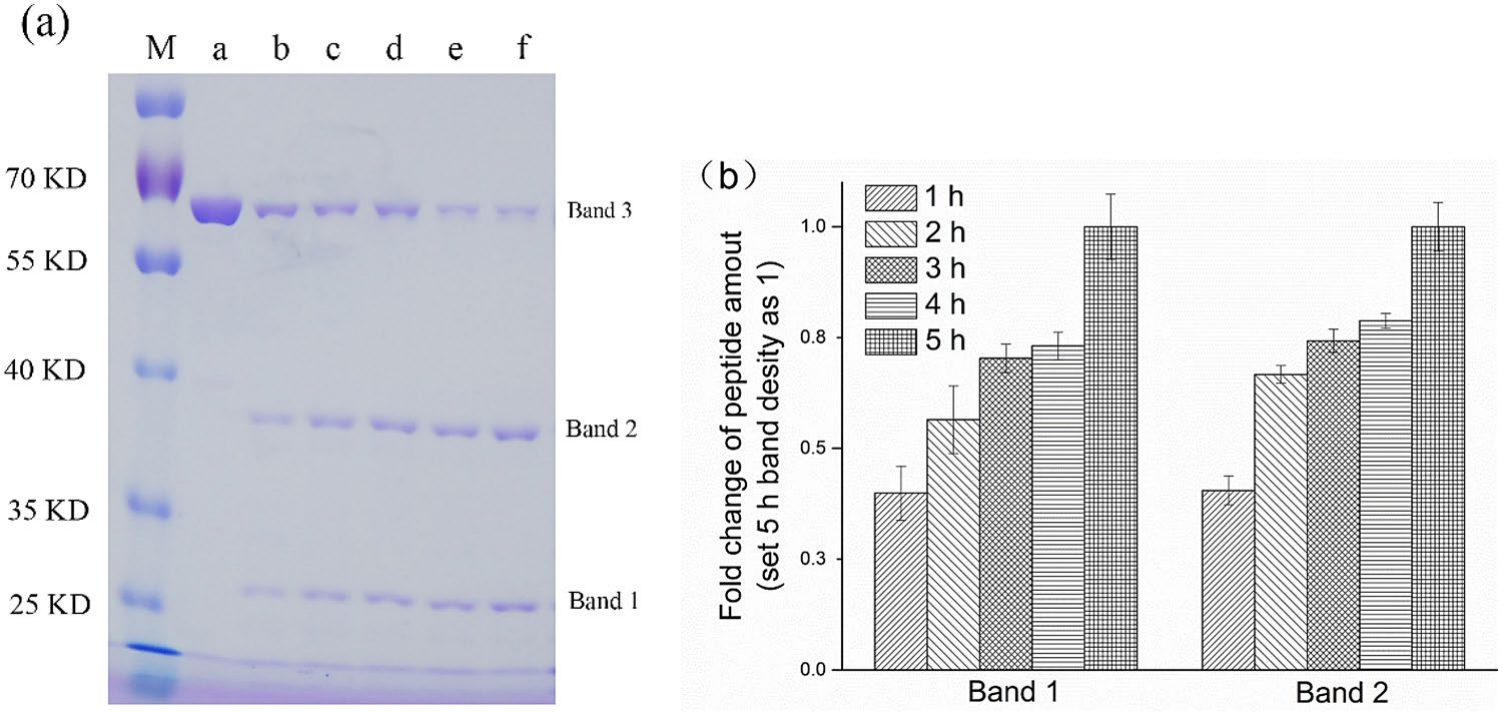
(**a**) Pronase enzymolysis results of Bt Cry1Ac protein. SDS image of pronase-digests of Bt Cry1Ac protein. Bt Cry1Ac (0.25 mg/mL) was digested with pronase (0.01 mg/mL) at incubation temperature of 37 °C for 0 (a), 1 (b), 2 (c), 3 (d), 4 (e) and 5 (f) hours. M, pre-stained protein ladders. Bands 1 and 2 are digest peptides of Bt Cry1Ac (band 3). (**b**) Densitometric analysis of peptides Band 1 and 2 of SDS-PAGE. Optical density was normalized to each 5 h Band’s value, respectively.

**Figure 2:**
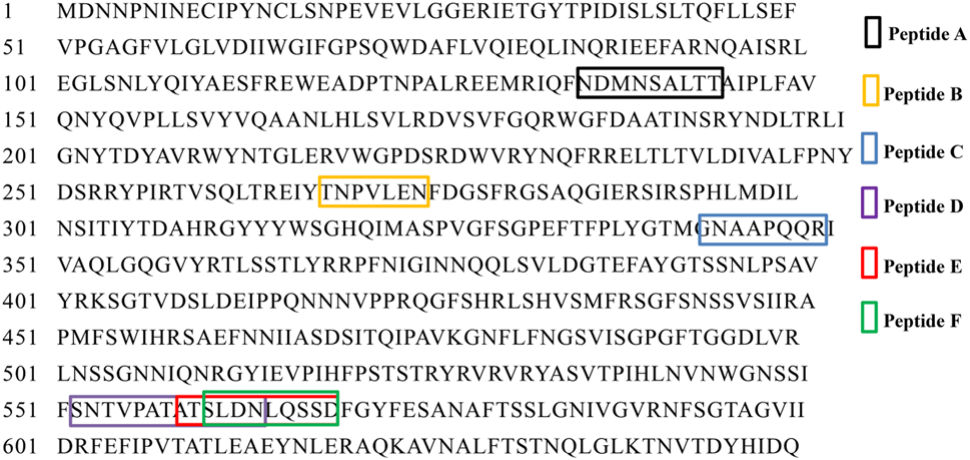
The locus of confirmed degraded peptides from Bt Cry1Ac protein. Peptides identified by nanoLC–MS/MS after Bt Cry1Ac was digested with pronase for 5 h at 37 °C. Peptide A (N^135^DMNSALTT^143^); peptide B (T^269^NPVLEN^275^); peptide C (G^342^NAAPQQR^349^); peptide D (S^552^NTVPATATSLDN^564^); peptide E (A^559^TSLDNLQSSD^569^); peptide F (S^561^LDNLQSSD^569^).

The SDS-PAGE image showed the Bt Cry1Ac protein was digested into band 1 and band 2 by pronase (Fig. 1a). The amounts of band 1 and band 2 enhanced with the increase of digestion time (0–5 h) (Fig. 1b). Both *N*-terminal amino acid sequencing and nanoLC–MS/MS analysis were used to confirm the degraded proteins (bands 1 and 2 in Fig. 1a). The peptides NH_2_-GINNQQLSVL-COOH and NH_2_-ETPYTPIDIS-COOH from bands 1 and 2, respectively, identically matched with the amino acid sequences of 374–383 and 30–39 of Bt Cry1Ac. In addition, after 5 h of digestion by pronase, peptides consisting about 10 amino acids were determined in triplicate by nanoLC MS/MS. Seven, eight and ten peptides in such size were identified in three technical replicates. Eleven peptides were identified (Table 1), and Peptide A, B, C, D, E, F were identified in all three technical replicates (Fig. 2).

**Table 1:** Peptides identified by nanoLC–MS/MS.

No	Sequence (*N–C*)	Number of amino acids	Molecular weight
Peptide A	F.NDMNSALTT.A	9	966.4
Peptide B	Y.TNPVLEN.F	7	786.4
Peptide C	M.GNAAPQQR.I	8	841.4
Peptide D	F.SNTVPATATSLDN.L	13	1290.6
Peptide E	T.ATSLDNLQSSD.F	11	1150.5
Peptide F	T.SLDNLQSSD.F	9	978.4
Peptide G	D.SLDEIPPQNN.N	10	1126.5
Peptide H	L.DEIPPQNNNVPPRQG.F	15	1674.8
Peptide I	F.SNTVPATAT.S	9	861.4
Peptide J	F.SNTVPATATS.L	10	948.5
Peptide K	Y.TNPVLENFDGS.F	11	1192.5

Pronase digestion sites were indicated with a period sign (.).

The digest yields of low-molecular-weight peptide were identified by nanoLC MS/MS in technical triplicate. Among the 11 peptides identified, peptides A–F were detected in all three replicates and peptides G–K were detected in one or two of the three replicates.

### Radioactive iodination

**Figure 3:**
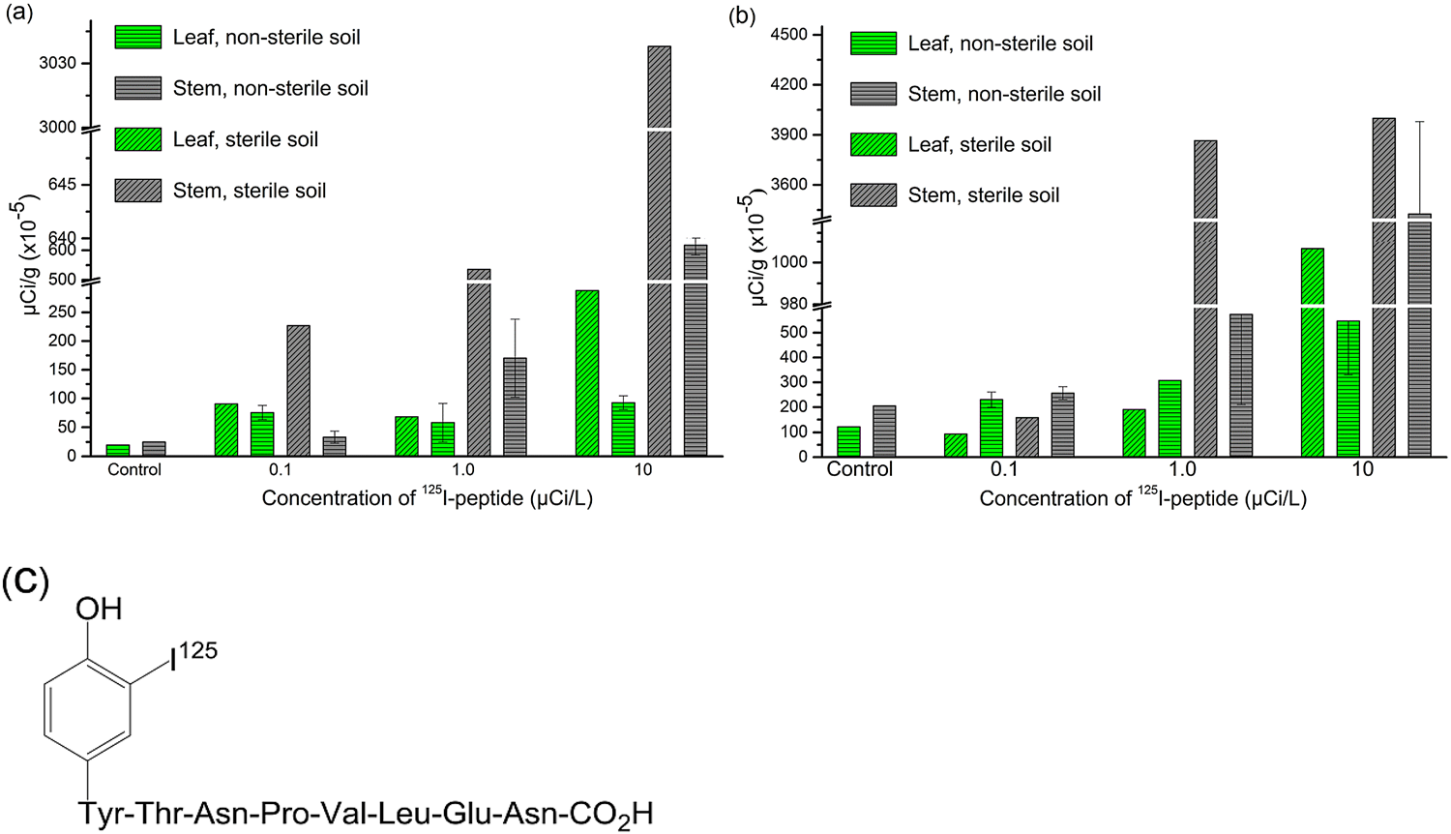
^125^I-peptide transferred from soil to leaves and stems. Absorption, uptake and translocation of ^125^I-labelled peptide YTNPVLEN (**c**) from soil to leaves and stems. Radioactivity value was detected in leaf (green) and stem (grey) of watermelon (**a**) and wheat (**b**) seedlings after application of ^125^I-peptide one hour later. Radioactivity value of plants cultivated in sterile soil (twill) was significantly higher than that in non-sterile soil (horizontal stripes).

The peptide B (NH_2_-YTNPVLEN-COOH) was radioactively iodinated (Fig. 3c). Two distinct radioactive peaks showed at retention times (Rt) of 4.969 and 7.838 min (Supplementary Fig. S1), which the former was unincorporated radioactive iodine and the latter was the iodinated peptide of interest. The radioactivity of the former and latter accounted for 46.4% and 53.6%, respectively, which indicated 53.6% of radioactive iodination yield and no side reactions.

### Transferability of peptide from soil to plant

The ^125^I-peptide was infiltrated into rhizosphere soil. Radioactivity in leaf and stem tissues of wheat and watermelon seedlings was detected at one hour after sprayings of the ^125^I-peptide (Fig. 3a,b), indicating that peptide B was transferred from soil to plants. In general, the radioactivity in wheat tissues was higher than that in watermelon and the intensity of radiation in stems of both wheat and watermelon was strong compared with that in leaves (Fig. 3a,b). The radioactivity level in their tissues after five hours of ^125^I-peptides fertilization was showed in Supplementary Table S3. Radioactivity in watermelon tissues was two- to ten-fold lower than that in wheat at the three radioactivity application levels (0.1, 1.0 and 10 μCi/L), except that the radioactivity in stems of watermelon and wheat was roughly similar at the 10 μCi/L level in the sterile soil. The ^125^I-peptide content in wheat tissues were in the magnitudes of 1.0 × 10^–15^ and 1.8 × 10^–14^ mol/g (fresh weight). The radioactivity of plants cultivated in sterile soil was significantly higher than that in non-sterile soil.

### Effect of peptides on soil fertility related bacteria and on physiology of wheat, watermelon and maize

**Figure 4:**
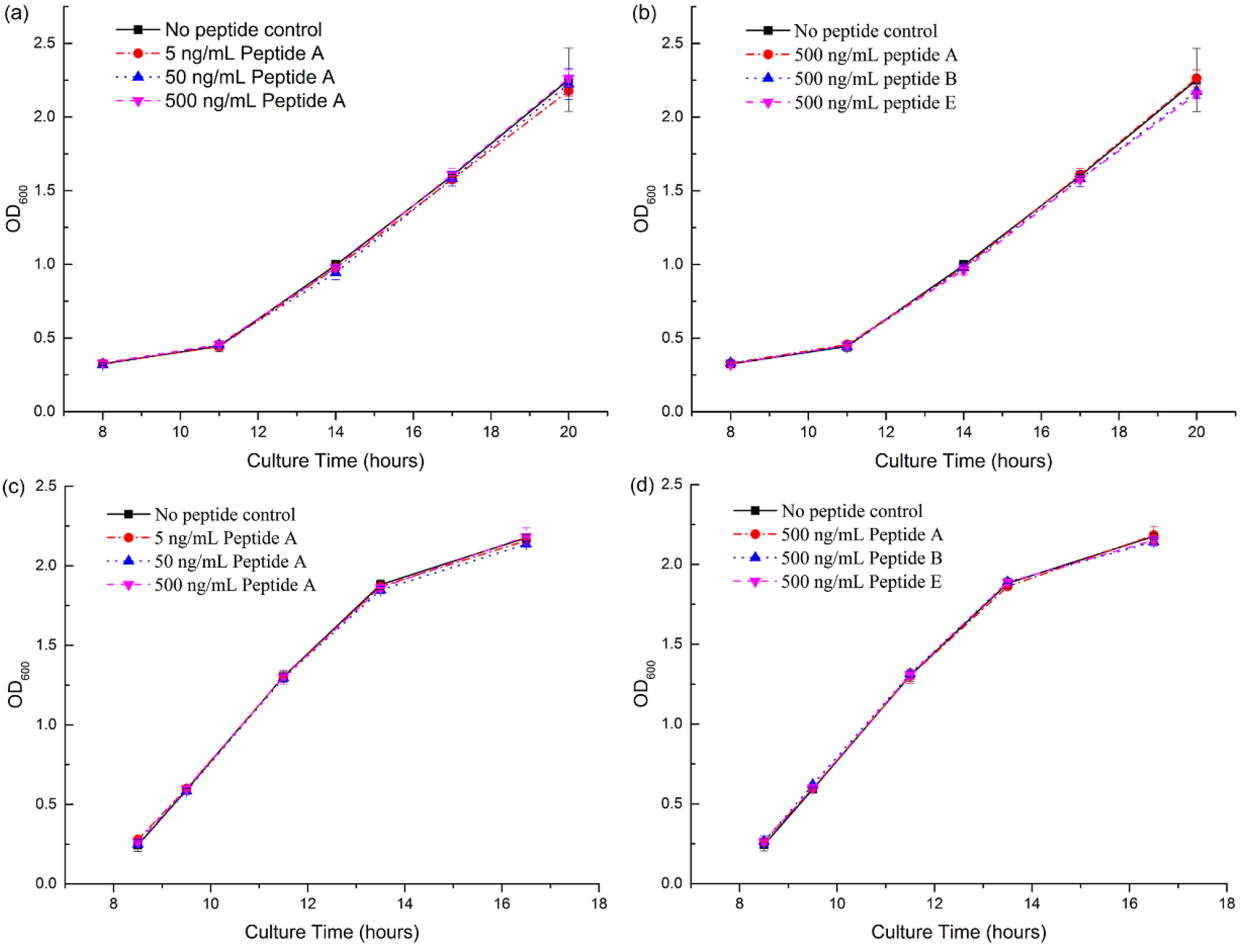
Effect of peptides on bacteria growth in liquid medium. Growth of organic phosphate-dissolving (OPD) bacteria (**a**,**b**) and nitrogen-fixing (NF) bacteria (**c**,**d**) by utilizing pronase-digested peptides (A, B, and E) supplemented in medium III (OPD bacteria) or medium V (NF bacteria). Every group has three replicates.

**Figure 5:**
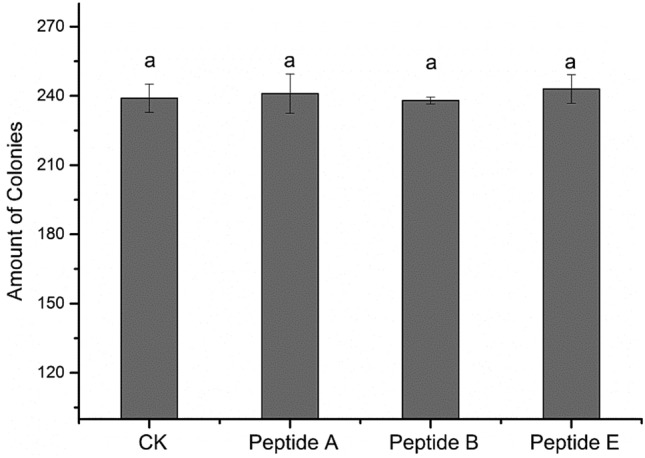
Colony number of potassium-dissolving bacteria after peptides treatment. Number of potassium-dissolving (PD) bacteria colonies after supplementation of the peptides A, B and E in medium IV. Every group had three replicates. The same letter indicated no statistical significance at P < 0.05 as determined by the LSD test.

The bacterial species, including phosphate-dissolving, nitrogen-fixing bacteria and potassium-dissolving bacteria, were isolated from soil and identified by alignment of 16S rRNA gene sequence (Supplementary Table S1). Peptides A, B and E at a concentration up to 500 ng/mL had no obvious effects on the growth of phosphate-dissolving and nitrogen-fixing bacteria (Fig. 4, data was showed in Supplementary Table S2) and potassium-dissolving bacteria (Fig. 5). In addition, those peptides had no significant effect on total soluble protein, total sugar, chlorophyll, superoxidedismutase and phenotype of watermelon, wheat and maize, except that chlorophyll b significantly increased by 9.1% in peptide B-treated watermelon (Table 2).

**Table 2:** Physiological indexes of watermelon, wheat and maize leaves after field application of peptides A, B and E.

Groups	Treatments	SOD (U/g)	Total soluble protein (mg/g)	Total sugar (mg/g)	Chlorophyll a (mg/g)	Chlorophyll b (mg/g)
Watermelon	Control	124.6a	8.31a	1.09a	1.31a	0.55b
	Peptide A	123.7a	8.42a	1.09a	1.34a	0.57ab
	Peptide B	126.5a	8.32a	1.10a	1.34a	0.60a
	Peptide E	121.5a	8.01a	1.08a	1.32a	0.55b
Wheat	Control	126.8a	3.86a	1.32a	1.42a	0.42a
	Peptide A	124.4a	3.82a	1.32a	1.42a	0.43a
	Peptide B	125.3a	3.81a	1.32a	1.41a	0.42a
	Peptide E	125.4a	3.83a	1.33a	1.41a	0.43a
Maize	Control	80.0a	11.50a	1.66a	2.33a	0.68a
	Peptide A	76.0a	11.02a	1.66a	2.37a	0.66a
	Peptide B	76.4a	11.73a	1.67a	2.46a	0.69a
	Peptide E	78.9a	11.30a	1.68a	2.34a	0.68a

All data were means of triplicate samples.

The same letter within a column indicated no statistical significance at P < 0.05 as determined by the LSD test.

## Discussion

The fate and risk of Bt protein have been controversial. The concentrations of Bt protein varied considerably in the rhizosphere of transgenic Bt crops during entire growth season^10^. Some studies indicated accumulation and persistence of Bt proteins in soil^5,35^. The other studies showed that Bt protein can be degraded soon in field soil and no accumulation after multiple years of transgenic crop use^7,8^. Many scientists found the risk of Bt protein to soil ecosystem was very significant^14,22^. The other studies suggested negligible risk of Bt protein in soil from Bt crops^2,15–17,19,20,23^.

Few studies on enzyme digestion of Bt proteins in soil have been reported^31^. Studies of the fate of Bt proteins in soil have had several challenges. Bt protein can be adsorbed and tightly bound on the clay minerals^35,36^, montmorillonite and kaolinite^28^, clay-size fraction and humic acids^6,12,13^. It is therefore difficult to extract Bt protein or its peptides from soil^23^. Substantial interfering substances in soil can cause interference problems^37^. A fortification study of pure Cry1Ac protein into soil would need a large amount of pure protein, which is costly for the fate study implementation. In addition, microbial degradation of Cry1Ac protein may vary largely due to microbial diversity and population dynamics at various environmental conditions^8,12,29–31^. In the present study, pure Bt Cry1Ac and proteolytic enzyme, produced by *Streptomyces griseus* that was prevalent in soil, were used to identify specific Bt peptides.

As a conservative estimate of the level of Cry1Ac protein was 650 ng/g^7^, the corresponding level of peptides was less than 5.9 × 10^–9^ M in soil (the average molecular weight of amino acids was 110 g/mol). In the bacteria growth experiment, the applied peptide concentration was 500 ng/mL, which was much greater than the reported concentration in soil. Our study initially revealed that no promoting or inhibiting effect of peptide hydrolyzed by Bt protein on the growth of soil functional bacteria. Peptides could also be nutrients for bacteria^28–30^, but the amount of added peptides in the medium was much less compared to the amount of peptone (1.0 × 10^7^ ng/mL), which are sources of carbon and nitrogen for bacterial growth. The amount of Bt protein and its degraded peptides was also relatively low in soil, which such small amounts of peptides might not affect the growth of bacteria as a nutrient.

^125^I has a half-life of 60 days and has been widely used in labeling peptides and proteins^38,39^. The shelf life of radiolabels varies somewhat among the peptides iodinated. Both radiation damage and spontaneous de-iodination probably contribute to the decreased radioactivity. Heber et al.^40^ discovered that the shelf life of radiolabels, ^125^I-labeled luliberin, corticotropin, calcitonin and parathyrin at 10 °C is 3–6, 3–4, 2 and 2 weeks, respectively. The aim of the present study was to verify if any peptides derived from Bt protein can be transferred to the intercrops of Bt-cotton. Watermelon, wheat and maize are the most common intercrops of Bt-cotton in China. Peptides containing tyrosine or histidine can easily undergo the iodine labeling reaction. In the Bt Cry1Ac protein amino acid sequence, the previous amino acid of peptide B is tyrosine. So tyrosine was added to the *N*-terminal of peptide B, and peptide YTNPVLEN was synthesized for ^125^I labeling. The maximal content of the ^125^I-peptide (^125^I-peptide solution of 10.0 μCi/L) was about 1.286 × 10^–16^ mol/cm^3^ in small-pots in the present study. This value was significantly smaller than the conservatively estimated level (5.9 × 10^–9^ M) of peptides deriving from Bt toxins in soil. In all treatment groups, notable radioactivity increase had been observed in both wheat and watermelon seedlings. These indicate that the metabolic peptides of Bt Cry1Ac from transgenic plant in soil could transfer to intercrops. The peptide transporters including oligopeptide transporters (OPTs) can transport di, tripe, tetra- and penta-peptides in plants^41^. The transporters of larger peptide (> 5 amino acids) are still unclear. The exogenously treatment of synthetic peptide RALF (5 kDa, ~ 50 amino acids) inhibited the root growth of tomato and *Arabidopsis thaliana*^42^. Similarly, exogenously supplied CLE peptides inhibited the formation of protoxylem vessels^43^. These results indicate that the larger peptide (> 5 amino acids) may be transferred from the surroundings to the plant, which is consistent with our results.

The ^125^I-peptide content in wheat and watermelon seedlings was about 1.0 × 10^–17^ to 1.0 × 10^–14^ mol/g. Radioactivity values in stems were larger than those in leaves commonly. The plants absorb nutrients from the roots, transport them to the stem, and then to the leaves. One hour after application, the ^125^I-peptide existed mainly in stems and a little has been transferred into leaves. In general, the radioactivity value in wheat tissues was higher than that in watermelon. This was because wheat seedlings had larger leaf surface area and more developed root system during the experimental stage. As the root pressure and leaf transpiration are the main impetus for nutrient transport and wheat seedlings had stronger transpiration and root pressure^44^, which resulted in the much higher radioactivity value in wheat seedling tissues.

The radioactivity value of plant cultivated in sterile soil was significant higher than that in non-sterile soil. The chemical and physical properties of sterile soil have been altered heavily^45–48^. Autoclaving soil has been shown to influence soil chemical properties^45^. The extractable Mn, N, P, S, NH_4_–N, NO_3_–N, organic matter, available P, water-soluble amino acids and carbohydrate levels increased significantly, but Al and Fe and trace element levels decreased in autoclaved soil^45,47,48^. Others have found that autoclaving can change the surface charge of pores in sandstone and reduce the surface area of clay^45–48^. The decrease of adsorption capacity of sterile soil particles may lead to easier translocation of peptides from soil into plant root. The soil sterilization can effectively kill microorganisms and inactivate all enzymes. This treatment was an effective method to prevent ^125^I-peptide from hydrolysis in soil in the present study.

Intercrop and rotation patterns have the potential to increase total system productivity, improve product quality, make full use of resources, resist pests, and enhance weed management and plant disease control^49,50^. Intercropping of cotton and wheat is a major application of multiple cropping, and plays an important role in combining food security and farmer’s income^25^. Intercropping of cotton and watermelon has been practiced in some area. In this study, peptides had no significant effect on main physiological indexes (total soluble protein, total sugar, superoxide and dismutase) of wheat, watermelon and maize except that peptide B has a significant increase on chlorophyll b content in watermelon seedlings. Plant peptides including systemin, clavata3/embryo surrounding region-related (CLE) peptides and rapid alkalization factor (RALF) act as key components of cell-to-cell communication^42,43^. These active signaling peptides regulate plant growth and development such as protoxylem vessel, stem cell differentiation and root growth and cause morphological change^42,43,51^. However, the phenotype of peptide B-treated watermelon seedling was not observed, which showed that peptide B maybe a non-bioactive peptide. Nakaminami et al.^52^ reported the external application of a hormone-like peptide AtPep3 (~ 30 amino acids) on *Arabidopsis* seedlings could affect the biosynthesis of chlorophyll. The chlorophyll content was significantly increased by 200–300% in comparison with the untreated control. While the chlorophyll b content was only increased by 9.1% in peptide B-treated seedlings, the effects of peptide B on chlorophyll b or other physiological indexes must be further verified. Identical peptide B (YTNPVLEN) sequence was also found in the ECF transporter S component (*Desulfosporosinus* sp. FKA, Genbank No. WP_088189292.1). However, the *C*-terminal of this peptide is leucine instead of phenylalanine in Bt Cry1Ac (YTNPVLENF).

In conclusion, Bt Cry1Ac protein was degraded into peptides by pronase secreted from *Streptomyces griseus*. The peptides can transfer into intercropping crops in the next season. The size or types of peptides that can be up taken by crops require further investigation. In general, the transferred peptides have shown no obvious influence on the crops or bacteria. This study provides a new view of GMO risk assessment methodology.

## Materials and methods

### Bacterial growth media

N-fixing bacteria were cultured with Waksman 77 medium (medium I, Glucose 10.0 g, MgSO_4_·7H_2_O 0.2 g, K_2_HPO_4_ 0.4 g, NaCl 0.2 g, 2 drops of 1% (*w/v*) FeCl_3_ and 1% (*w/v*) MnSO_4_ solution, 1% (*w/v*) Congo Red solution 5 mL, agar 18 g, distilled water 1000 mL, pH 7.0.). Organic phosphate-dissolving bacteria were cultured with Menkina medium (medium II, Glucose 10.0 g, (NH_4_)_2_SO_4_ 0.5 g, NaCl 0.3 g, KCl 0.3 g, MgSO_4_·7H_2_O 0.3 g, FeSO_4_·7H_2_O 0.03 g, MnSO_4_·4H_2_O 0.03 g, lecithin 0.2 g, CaCO_3_ 5.0 g, agar 18.0 g, distilled water 1000 mL, pH 7.0–7.5.) and liquid medium (medium III, Glucose 10.0 g, (NH_4_)_2_SO_4_ 0.5 g, NaCl 0.3 g, KCl 0.3 g, MgSO_4_·7H_2_O 0.3 g, FeSO_4_·7H_2_O 0.03 g, MnSO_4_·4H_2_O 0.03 g, lecithin 0.2 g, CaCO_3_ 5.0 g, yeast extract 0.4 g, distilled water 1000 mL, pH 7.0–7.5.). Potassium-dissolving bacteria were cultured with Aleksandrov’s medium (medium IV, Sucrose 5.0 g, MgSO_4_·7H_2_O 0.5 g, CaCO_3_ 0.1 g, Na_2_HPO_4_·12H_2_O 2.0 g, FeCl_3_·6H_2_O 0.005 g, potassium-bearing rock powders 1.0 g, agar 18.0 g, distilled water 1000 mL, pH 7.0–7.5) and Beef extract-peptone medium (medium V, Beef extract 3.0 g, peptone 10.0 g, NaCl 1.0 g, distilled water 1000 mL, pH 7.0–7.2.). All of the above media were autoclaved at 115 °C for 30 min.

### Enzymolysis of Cry1Ac protein and identification of small peptides

One mg of Bt Cry1Ac protein (Youlong Biotech LLC, Shanghai, China) was dissolved in 4.0 mL phosphate buffer solution (PBS, 10 mM, pH 7.4), followed by addition of 400 μL pronase solution (0.1 mg/mL, Sigma-Aldrich, St. Louis, MO, USA). After 0, 1, 2, 3, 4 and 5 h of incubation at 37 °C, an aliquot of enzymatic hydrolyzate was sampled for sodium dodecyl sulfate polyacrylamide gel (SDS-PAGE, 12%) analysis. After enzyme hydrolysis for 5 h, the pH of buffer was adjusted to 2.0 with 2 M HCl to deactivate pronase. The enzymatic digest was filtered at a molecular weight cutoff of 3000 Da (Merck Millipore, Germany) to remove large-molecular-weight peptides. The filtrate was collected in a tube and then mixed with 5.0 mL of aqueous formic acid (FA, 0.1%, v/v). The mixture was desalted with a C_18_ desalination column, lyophilized and then reconstituted in 300 μL 0.1% FA for nano liquid chromatography mass spectrometry analysis (nanoLC–MS/MS)^53^.

The nanoLC–MS/MS analyses were performed on a nano liquid chromatograph (Proxeon Biosystems) Q-Exactive mass spectrometer (Thermo Finnigan) system. The peptide mixtures were pre-concentrated onto a Thermo scientific EASY C_18_ column (2 cm × 100 μm, 5 μm) and separated on a Thermo scientific EASY C_18_ column (100 mm × 75 μm, 3 μm) by gradient elution. The mobile phase consisted of 0.1% FA (solution A) and 84% aqueous acetonitrile containing 0.1% FA (solution B) and programmed by increasing solution B from 0 to 50% (v/v) in 50 min, to 100% in 4 min, and then remained at 100% for 6 min. The flow rate was 250 nL/min.

The mass spectrometer was operated in positive ion mode and higher-energy collisional dissociation (HCD) by using a data-dependent top 10 method to dynamically choose the most abundant precursor ions (300–1800 *m/z*) from the survey scan. Automatic gain control (AGC) target was set at 3e6, and maximum inject time at 10 ms. Determination of the target value was based on predictive AGC. Dynamic exclusion duration was 20.0 s. Survey scans were acquired at a resolution of 70,000 at *m/z* 200 and resolution for HCD spectra was set at 17,500 at *m/z* 200, and isolation window was set at 2 *m/z*. MS/MS maximum inject time was set at 60 ms. Normalized collision energy was 27 eV and the underfill ratio, which specified the minimum percentage of the target value likely to reach at maximum fill time, was defined as 0.1% on the Q-Exactive. The instrument was run in peptide recognition mode.

Raw MS/MS spectral data were searched using Mascot engine (Matrix Science, London, UK; version 2.2) against a Bt Cry1Ac protein amino acid sequence database. For peptide identification, the following options were used: Enzyme at none, and two missed cleavages permitted. The precursor mass tolerance was set at 20 ppm, MS/MS tolerance was set at 0.1 Da. The fixed modification was carbamidomethyl for cysteines, and the variable modification was oxidation for methionine. The false discovery rate (FDR) for peptides and modification site was set at 0.01. A MaxQuant score was set at ≥ 20.

### The localization of large peptides

Both *N*-terminal amino acid sequencing and nanoLC–MS/MS analysis were used to confirm the large peptides. The *N*-terminal amino acid sequences of large peptides (bands 1 and 2 in Fig. 1a) in gel were analyzed by Edman method^54^. The PVDF membrane (Millipore, USA) was immersed in absolute methanol for 10 s followed by rinsing in distilled water and equilibration in transfer buffer (10 mM CAPS, 10% methanol, pH 11.0). The SDS-PAGE gels were blotted at 250 mA for 1.0 h using the DALT Blotting Kit (Bio-Rad, USA). The PVDF blots were then washed twice by distilled water, stained with Coomassie brilliant blue (0.05% Coomassie brilliant blue R-250, 50% methanol, 9.2% acetic acid in distilled water) for 45 min, and destained in absolute methanol for 1.0 min.

The protein spots were cut from dry PVDF membrane and placed in small tubes. 600 µL 0.1% trifluoroacetic acid was added into every tube. Then the tubes were incubated in a thermostatic shaker (room temperature, 600 rpm) for 1.0 min, removing the supernatant, repeated 3 times. The membranes were air-dried, cut into 0.5 cm^2^ in size and then loaded in the blot cartridge. The sequences were analyzed on an automatic peptide sequencing machine (PPSQ-33A, Shimadzu) at APTbiotech (Shanghai, China).

### Radioactive iodination of the octa amino acid peptide YTNPVLEN

To track the translocation of derived peptide, synthetic peptides (SBS Genetech Co., Ltd, Beijing, China) are labeled with radioactive elements. ^125^I is commonly used for labelling peptides on tyrosine^55,56^. There was a tyrosine previous to the *N*-terminal of peptide B (H_2_N-TNPVLEN-COOH) in the Bt Cry1Ac amino acid sequence. So peptide H_2_N-YTNPVLEN-COOH was synthesized for iodination labelling by chloramine-T method^39^ at the State Key Laboratory of Radioactive Chemical Drugs of Beijing Normal University. An aliquot of 100 µL of chloramine-T (0.99 µg/µL) and 1.0 mCi of Na^125^I (18.5 MBq) were added into 2.5 mL of peptide solution (40 μg/mL). After stirred for 5 min at room temperature, 100 µL of sodium pyrosulfite (Acfa Aesar, Beijing, China) solution (1.67 µg/µL) was added to stop the reaction. The mixture was loaded on SCL-10Avp high performance liquid chromatograph (Shimadzu, Japan), separated by a semi-preparative Venusil MP C-18 column (10 mm × 250 mm, Bonna-Agela Technologies). The radiolabeled peptide was eluted with acetonitrile at a flow rate of 2.0 mL/min and monitored by a UV detector (254 nm). The fraction at the retention time period of 7.6–8.3 min was collected. The radioactivity was determined by a Gamma Counter (Wallac, USA). The ^125^I-peptide was purified for the following experiment.

### Cultivation of crop seedlings

The soil, in which non-transgenic crops were previously grown, was collected from China Agricultural University’s experimental field (N 40° 01′, E 116° 16′) at a depth of 0–15 cm. The soil had a pH of 7.9 and was consisted of 48.8% sand, 48.2% silt, 3.0% clay, 2.6% organic matter, 0.16% total nitrogen, 60.1 mg/kg available phosphorus and 95.5 mg/kg available potassium. It was air-dried at room temperature and used to grow plant seedlings.

Wheat and watermelon seeds were immersed in sterile deionized water at 37 °C for 5 h and 10 h, respectively. The seeds were spread evenly on the germinating paper (25 × 38 cm) (OR 97321, Hoffman Manufacturing Inc., Albany, USA) in germination boxes. The wheat and watermelon seeds were sprouted 4 and 2 days, respectively, in a DRX-450C growth chamber (Ningbo Safe LLC., Zhejiang, China) set at 28 ± 1 °C and humidity at 70 ± 5% (12 h/12 h, light/dark circular). After that, the seedlings were transplanted into small pots (350 cm^3^, 20 seedlings per pot), which contained sterile or non-sterile soil. The soil sterilization process was as follows: the soil powder was tiled in an aluminum box, its depth was approximately 2 cm. They were autoclaved at 121 °C for 3 h, and were dried at 103 °C for 3 h^45^. The pots were kept in the growth chamber 6 days for watermelon and 5 days for wheat.

### Fertilization of ^125^I-peptide and measurement of radioactivity

Six days after wheat seedlings and 5 days after watermelon seedlings transplant, the seedlings were respectively fertilized with ^125^I-peptide solution. Sixteen pots of watermelon and wheat seedlings, respectively, were divided into four groups. The first group which fertilized with isometric sterile deionized water was served as the control group. The other three treatment groups were added with 10.0 mL ^125^I-peptide solution of 0.1, 1.0, 10.0 μCi/L, respectively. In each group, one pot was with sterile soil and other three pots were with non-sterile soil. The radioactive solution was squirted out from an injector to the surface of soil to make sure that no solution was adhered to plant surface. Those seedlings were cultivated in the growth chamber set at 25 ± 1 °C, and 10 h/14 h of light/dark circular.

At set intervals, one plant was sampled from every small-pot after fertilization. The leaves and stems were cut off and weighed, respectively. The radioactive intensity of those tissues was measured by WIZARD2 Automatic Gamma Counter for 2 days. All data were recorded automatically.

### Effect of peptides on soil functional bacteria

Colonies of organic phosphate-dissolving bacteria, nitrogen-fixing bacteria and potassium-dissolving bacteria were isolated from the soil of experimental field as previously described^10,57^ and the colonies were stored at 4 °C until further use. Bacterial species were identified by alignment of partial 16S rRNA gene sequences against the GenBank database^36,58^. The identification criteria described by Joseph^59^ were adopted.

Organic phosphate-dissolving bacteria were activated in beef extract-peptone medium. The bacteria were cultured in a thermostatic shaker (JC-211B, Jingchang Yiqi, Shanghai, China) at 28 °C under shaking 160 rpm. The optical density (OD, λ = 600 nm) of the culture was measured with a UV-2550 spectrophotometer (Shimadzu, Suzhou, China). After OD_600_ of the medium reached to 1.0, 40 µL of the bacteria was transferred into 40 mL of liquid medium (medium III). Meanwhile, the synthesized peptides A, B and E were added in medium to a series of final concentrations of 0, 5, 50, 500 ng/mL. Every group was in triplicate. The bacteria were cultured on the thermostatic shaker again. The procedure of bacteria activation and peptides addition for culturing nitrogen-fixing bacteria was the same as organic phosphate-dissolving bacteria, while beef extract-peptone medium was used for bacteria growth evaluation study. OD_600_ values were determined to evaluate the effect of peptides on the bacterial growth.

The peptides addition for culturing potassium-dissolving bacteria was the same as above. The final concentration of the three peptides was all 500 ng/mL in solid Aleksandrov’s medium. Every group was in triplicate. A colony was dissolved in sterile distilled water to an appropriate concentration, and 100 μL of the dilution was used to inoculate each plate. All plates were incubated in growth chamber at 28 °C. Four days later, the number of strain colonies was counted. The effect of peptides on potassium-dissolving bacteria was evaluated based on the number of colonies.

### Effect of peptides on wheat, watermelon and maize physiology

Wheat seedlings in small pots were cultivated in the DRX-450C growth chamber set at 28 ± 1 °C and humidity at 70 ± 5% (12 h/12 h, light/dark). Twenty-four pots of wheat seedlings were divided into four groups. When the height of seedlings was about 15 cm, each of the three treatment groups was sprayed 2.0 mL of 1.0 μg/mL of peptides (A, B and E) solution, while the control group was sprayed with the same volume of sterile deionized water. The spraying was repeated one week later. Watermelon seedlings and maize (*Zea mays* L.) seedlings were planted at Shangzhuang Experimental Field (N 40° 08′, E 116° 10′, Beijing, China). When the length of watermelon vines was about 30 cm and the height of maize stalks was about 1.2 m, each watermelon and maize plant was sprayed 4.0 mL of 1.0 μg/mL peptides (A, B and E) solution.

One week after the second spraying, the leaves were collected and stored at − 40 °C before further study. The physiological indexes of total soluble protein, total sugar, chlorophyll and SOD were determined as described previously^8,60–62^.

### Statistical analyses

To assess the effect of peptides on bacteria and crops, the OD_600_ value of bacterial medium, the numbers of bacterial colonies and physiological indexes of crops were statistically analyzed with Duncan’s Multiple Range Test at a significance level of 5% in SPSS 17 (Chicago, IL, USA).

## Supplementary information

Supplementary Information.
