# Supplementary material for: Absorption, translocation, and effects of Bt Cry1Ac peptides from transgenic cotton to the intercrops and soil functional bacteria
Source: Sci Rep. 2020 Oct 14;10:17294. doi: 10.1038/s41598-020-73375-8 (PMC7557920; doi:10.1038/s41598-020-73375-8)
Supplement: Supplementary file 1 — Supplementary Information. [file 41598_2020_73375_MOESM1_ESM.pdf]

Supplementary information

Absorption, translocation, and effects of Bt Cry1Ac peptides from transgenic cotton to the intercrops and soil functional bacteria

Short title: Effects of Bt Cry1Ac peptides to the intercrops

Wei Zhang[+], Zhen Cao[+], Mian Wang, Xiaojiao Chen*, Baomin Wang*

College of Agriculture and Biotechnology, China Agricultural University, Yuanmingyuan west road No.2, Beijing 100193, China

* Correspondence author:

B. Wang, phone: +86-10-62731305. Fax: +86-10-62732567. E-mail: wbaomin@263.net

X. Chen, phone: +86-10-62731305. Fax: +86-10-62732567. E-mail: chenxj913@126.com

+These authors contributed equally to this work.

Table S1. The analysis results of 16S rRNA gene sequences of nitrogen-fixing bacteria, potassium-dissolving bacteria and organic phosphate-dissolving bacteria

Name	Reference species	GenBank Accession	Class and subclass	16S rRNA homology
potassium-dissolving bacteria	*Oxalobacteraceae bacterium*	DQ388765	Bacteria; Proteobacteria; Betaproteobacteria; Burkholderiales; Oxalobacteraceae.	99%
nitrogen-fixing bacteria	*Azotobacter chroococcum*	JQ692178	Bacteria; Proteobacteria; Gammaproteobacteria; Pseudomonadale; Pseudomonadaceae; Azotobacter	100%
organic phosphate-dissolving bacteria	*Pseudomonas protegens*	KM589021	Bacteria; Proteobacteria; Gammaproteobacteria; Pseudomonadale; Pseudomonadaceae; Pseudomonas.	100%

Table S2. The effect of peptides on the growth of nitrogen-fixing bacteria and organic phosphate-dissolving bacteria

Bacteria	Groups	Spiked concentration (ng/mL)	Stagnant growth phase	Logarithmic growth phase	Stationary growth phase
nitrogen-fixing bacteria	Control group	0	0.26±0.038 a	1.88±0.031 a	2.18±0.019 a
	Peptide A	5	0.28±0.014 a	1.87±0.016 a	2.16±0.012 a
		50	0.25±0.017 a	1.85±0.023 a	2.14±0.016 a
		500	0.26±0.006 a	1.86±0.017 a	2.18±0.055 a
	Peptide B	5	0.28±0.021 a	1.89±0.005 a	2.17±0.019 a
		50	0.28±0.029 a	1.87±0.038 a	2.17±0.133 a
		500	0.26±0.036 a	1.89±0.008 a	2.14±0.027 a
	Peptide E	5	0.27±0.022 a	1.91±0.076 a	2.16±0.044 a
		50	0.26±0.048 a	1.89±0.057 a	2.23±0.125 a
		500	0.26±0.022 a	1.89±0.012 a	2.15±0.027 a
organic phosphate-dissolving bacteria	Control group	0	0.32±0.008 a	1.60±0.009 a	2.25±0.214 a
	Peptide A	5	0.33±0.018 a	1.58±0.019 a	2.18±0.036 a
		50	0.32±0.008 a	1.58±0.05 a	2.22±0.105 a
		500	0.33±0.005 a	1.61±0.044 a	2.26±0.058 a
	Peptide B	5	0.32±0.011 a	1.58±0.038 a	2.26±0.079 a
		50	0.33±0.003 a	1.58±0.042 a	2.21±0.038 a
		500	0.33±0.012 a	1.58±0.053 a	2.18±0.045 a
	Peptide E	5	0.33±0.015 a	1.59±0.023 a	2.22±0.082 a
		50	0.33±0.014 a	1.57±0.026 a	2.14±0.106 a
		500	0.32±0.007 a	1.58±0.019 a	2.16±0.029 a

All data were means of triplicate samples.

The same letter within a column indicated no statistical significance at $P < 0.05$ as determined by the LSD test

Table S3. The radioactivity level in the tissues of watermelon and wheat seedlings after 5 hours of ^{125}I-peptides fertilization

^{125}I-pepetide concentration	Soil treatment	Radioactivity level/ µCi/g (x 10^{-5})			
		Watermelon leaf	Watermelon stem	wheat leaf	Wheat stem
Control group	non-sterile soil	19.6	25.2	120.4	204.7
0.1 µCi/L	sterile soil	84.4	104.4	193.1	628.3
	non-sterile soil	101.7	107.0	154.8	537.5
		37.6	68.5	217.0	450.3
		58.5	46.9	169.1	454.7
1 µCi/L	sterile soil	178.4	579.3	849.0	3704.1
	non-sterile soil	71.5	142.1	442.0	931.0
		63.4	59.5	187.5	452.7
		146.4	156.1	141.9	374.0
10 µCi/L	sterile soil	840.6	10229.7	6626.2	33360.0
	non-sterile soil	103.3	275.5	1140.3	5689.9
		91.6	257.1	2002.7	4130.8
		115.5	253.9	848.8	1835.4

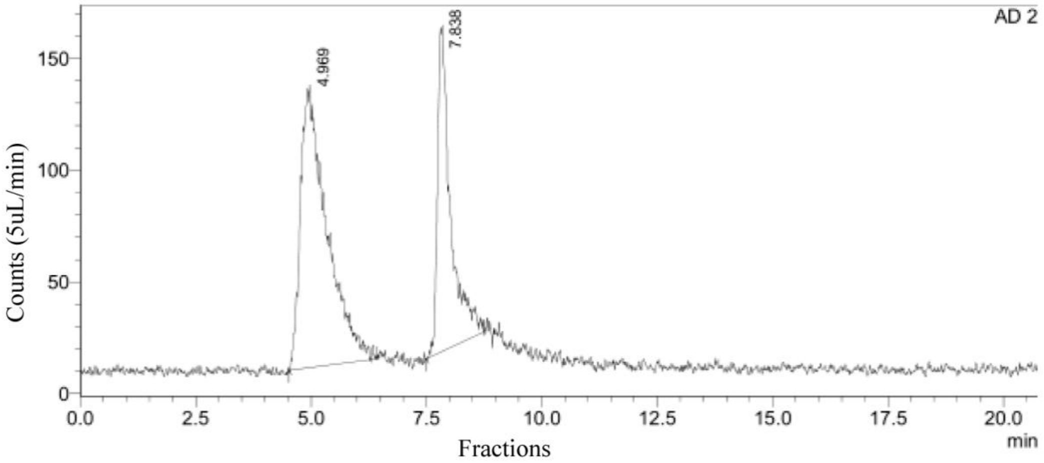

Figure S1. Radiochromatogram of ^{125}I-labeled NH_2-YTNPVLEN-COOH forms separated by HPLC. Continuous line indicates radioactivity values of fractions taken. The first peak represents the activity of unincorporated iodine (retention time: 4.969 min), the second peak (retention time: 7.838 min) shows activity of the iodinated peptide form.
